# Immunomodulating Hydrogels as Stealth Platform for Drug Delivery Applications

**DOI:** 10.3390/pharmaceutics14102244

**Published:** 2022-10-21

**Authors:** Zahra Rezaei, Dilara Yilmaz-Aykut, Fatima Mumtaza Tourk, Nicole Bassous, Margot Barroso-Zuppa, Asif Iqbal Shawl, Syed Salman Ashraf, Huseyin Avci, Shabir Hassan

**Affiliations:** 1 Division of Engineering in Medicine, Brigham and Women’s Hospital, Department of Medicine, Harvard Medical School, Cambridge, MA 02139, USA; 2 Chemical Engineering Department, Sharif University of Technology, Azadi Ave, Tehran 11365-11155, Iran; 3 Department of Chemical Engineering, Faculty of Engineering, Istanbul University-Cerrahpaşa, 34320 Istanbul, Turkey; 4 Department of Mechanical Engineering, Northeastern University, Boston, MA 02115, USA; 5 School of Medicine and Health Sciences, Tecnologico de Monterrey, Mexico City 14380, Mexico; 6 Department of Biology, College of Arts and Sciences, Main Campus, Khalifa University, Abu Dhabi P.O. Box 127788, United Arab Emirates; 7 Center for Biotechnology (BTC), Main Campus, Khalifa University, Abu Dhabi P.O. Box 127788, United Arab Emirates; 8 Center for Catalysis and Separation (CeCas), SAN Campus, Khalifa University, Abu Dhabi P.O. Box 127788, United Arab Emirates; 9 Advanced Materials Chemistry Centre (AMCC), SAN Campus, Khalifa University, Abu Dhabi P.O. Box 127788, United Arab Emirates; 10 Department of Metallurgical and Materials Engineering, Eskisehir Osmangazi University, 26040 Eskisehir, Turkey; 11 Cellular Therapy and Stem Cell Research Center, Eskisehir Osmangazi University, 26040 Eskisehir, Turkey; 12 Translational Medicine Research and Clinical Center, Eskisehir Osmangazi University, 26040 Eskisehir, Turkey

**Keywords:** hydrogels, immune modulation, biomaterials, foreign body response, tissue engineering

## Abstract

Non-targeted persistent immune activation or suppression by different drug delivery platforms can cause adverse and chronic physiological effects including cancer and arthritis. Therefore, non-toxic materials that do not trigger an immunogenic response during delivery are crucial for safe and effective in vivo treatment. Hydrogels are excellent candidates that can be engineered to control immune responses by modulating biomolecule release/adsorption, improving regeneration of lymphoid tissues, and enhancing function during antigen presentation. This review discusses the aspects of hydrogel-based systems used as drug delivery platforms for various diseases. A detailed investigation on different immunomodulation strategies for various delivery options and deliberate upon the outlook of such drug delivery platforms are conducted.

## 1. Introduction

One of the most concerning issues with conventional drug delivery platforms is the elicitation of an immune response upon implantation [1,2]. Different natural and artificial platforms have been used for various biomedical applications ranging from drug and metabolite delivery [3], gene delivery [4], and wound healing/regenerative applications [5,6]. However, most of these platforms suffer due to a compromise on immunogenicity and their respective biomedical applications. Although hydrogels from biomaterials of different origins have shown great promise in various biomedical applications, their immunogenicity, however small, is still a matter of concern, thus preventing their widespread clinical adoption [7]. Hydrogels have been proposed as an excellent platform for various applications in drug delivery and regenerative medicine. Hydrogels are soft, tridimensional crosslinked networks of polymers with a high-water content [8,9,10,11,12], similar to the percentage found in human tissue [9]. Due to their hydrophilic nature, hydrogels can act as biological nests and mimic the nature of tissue by being permeable to oxygen, ions, nutrients, metabolites, and waste products [11,12]. Moreover, hydrogels have demonstrated good biocompatibility, controllable stiffness for cell culture, and design flexibility, putting them in the spotlight in the research fields of biomaterials and immunomodulation over the past few decades [8,10,11]. They offer the ease of controlling immune responses by surface modulation via chemisorption/release of different adjuvants, mechanical properties, tissue adhesion, and the addition of immune-suppressing biomolecules [13]. Thanks to their potential to build an environment that supports transplanted cells and to re-establish the lost function of tissues due to disease, immunomodulating hydrogels (IMHs) offer plenty of promising medical applications, especially in tissue regeneration [14,15], cancer treatment [16,17], and inflammation control [18,19]. In this review, we touch upon some of the exciting opportunities hydrogels present in the form of versatile drug delivery platforms for treatment and regenerative medicine. We review several studies where IMHs were used to address immunogenicity issues that hamper their exploitation for various biomedical tasks. While the field is attracting a lot of research and holds great promise, there is still a lacuna in the field that will lead to widespread adoption of immune modulating hydrogels for drug delivery and long-term regenerative applications. In addition to the various immune modulation strategies for hydrogels, we briefly discuss some of the shortcomings that need to be addressed before their full potential can be realized. This critical review also provides guidance on the different types of immunomodulating molecules that can act as three-dimensional biomedical cargo carriers. Moreover, it also discusses how IMHs can be used to control inflammation in cancer and diabetes treatment as well as for tissue repair and regeneration applications.

### 1.1. Tissue Regeneration and Inflammation Control

**Figure 1:**
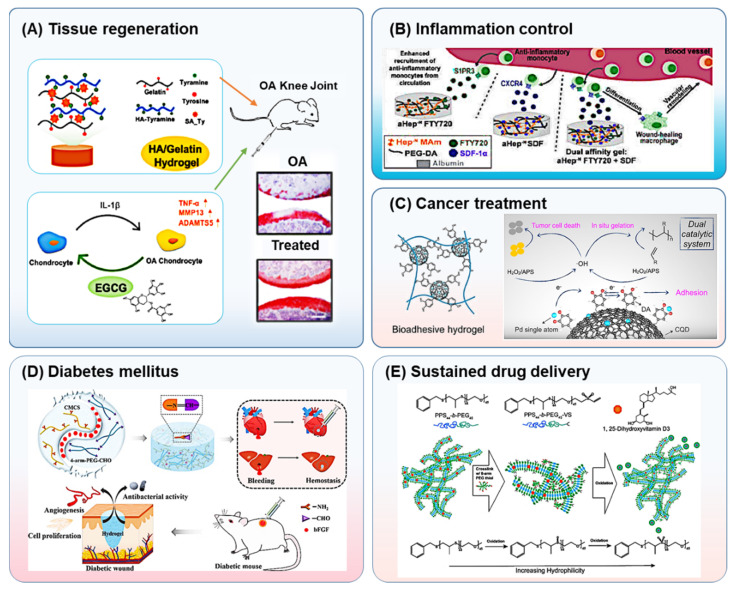
Different applications of immunomodulating hydrogels (IMHs) in disease treatments. (**A**) Tissue regeneration: Osteoarthritic (OA) cartilage repair. IMHs promote tissue regeneration and inflammatory cascade inhibition alongside drug delivery (redrawn from the original article [25]). (**B**) Inflammation control: Heparin-loaded hydrogels modulate inflammation and fibrosis incidence in medical implants (redrawn from the original article [20] (**C**) Cancer therapy: immunomodulating injectable hydrogel with carbon quantum dot for localized cancer immunotherapy (redrawn from the original article [31]. (**D**) Diabetes mellitus: Using chitosan-based hydrogel containing bFGF for diabetic wound healing. (**E**) Sustained release drug delivery: one type of vitamin D delivery. Hydrogel oxidation causes changing the hydrophobic/ hydrophilic ratio and a morphological transition in micelles’ structures resulting in release (Adapted with permission from [32]. Reproduced with permission from Elsevier (**A**,**C**,**D**), American Chemical Society (**B**), and Frontiers in Bioengineering and Biotechnology (**E**).

Current biomedical implants and tissue engineering technologies show a lot of promise to improve the function of unhealthy organs. Nevertheless, one of the daunting challenges to overcome for true clinical success is the lack of functional grafting of implants [20]. Acute/chronic inflammation is a significant issue that affects implantation via different immune components, primarily monocytes from the host, which can develop into macrophages or foreign body giant cells [21]. It is well-established that the phenotype of macrophages can change during inflammation from anti-inflammatory to pro-inflammatory. The pro-inflammatory macrophages migrate to the injury site and stimulate the healing process. In contrast, the anti-inflammatory macrophages help immune regulation for tissue repair and remodeling [22,23]. Any imbalance in this immune response can wreak havoc in the form of autoimmune diseases such as osteoarthritis and diabetes. Osteoarthritis is one of the most prominent bone diseases linked to old age, obesity, and traumatic injury. This is a disabling and degenerative disease that affects synovial joints and causes cartilage deterioration. It results in joint inflammation and drastically affects the lifestyle of the afflicted [24]. A hydrogel containing bioactive glass has been reported to promote both hard (bone) and soft (skin) tissue regeneration [25]. Thus, IMHs can assist treatment through catabolic pathways and inflammatory cascade inhibition alongside chondrogenesis promotion ability and drug delivery (Figure 1A) [26]. While macrophages are essential in tissue regeneration and repair, their presence in diabetic wounds preserves a persistent pro-inflammatory phenotype that hinders the healing process. Therefore, modulating macrophages in diabetic wounds is another promising strategy involving drug- [27], cell- [28], or exosome- [29] loaded hydrogel application in promoting healing. Hydrogel scaffolds can protect the islet cell transplant in the same way that the drug delivery process does. In yet another study, proper vascularization in salivary gland regeneration was achieved by taking advantage of both the drug, such as deferoxamine, and immune modulation properties via laminin- and growth factor-conjugated hydrogels [30].

Hydrogels can control inflammation via different mechanisms [28], such as (1) releasing agents that prevent the migration of monocytes or maturation of macrophages, (2) hindering macrophage infiltration, (3) scavenging harmful products from activated macrophages like reactive oxygen species, (4) promoting an M2-like macrophage phenotype, (5) preventing the migration of activated T cells from lymph nodes, and (6) inducing immune cell apoptosis.

Another important aspect of immunogenicity to any foreign material is the effects after treatment. While inflammation progression in the body can be lessened by different mediators, it leaves scars in response to injury or damage resulting in the formation of fibrous connective tissue at the healing site [33]. Utilizing immunomodulation materials, like mucin [34] or heparin (Figure 1B) [21], hydrogels have been shown to considerably modulate fibrosis incidence in medical implants and tissue engineering therapies.

### 1.2. Cancer Treatment

Hydrogels loaded with tumor-suppressing cells such as T cells, and other relevant drugs can be applied adjacent to or at tumor regions for effective drug delivery and dispersion [16]. For example, hydrogel doped with anti-CD47 antibodies can promote phagocytosis by modulating the expression of the CD47 protein on the tumor surface [35]. Another interesting approach for cancer treatment is the use of targeted drug delivering hydrogels to provide localized cancer treatment. To do so, biomacromolecular drugs such as antibodies antigens, nucleic acids, and proteins are loaded in hydrogels along with the therapeutic drug. 

Apart from providing an amiable delivery route, hydrogels also positively affect the half-life of the encapsulated drugs that may be labile and degrade rapidly [36]. Hence, hydrogels can contribute to the better availability of drugs by making them available for a longer time and releasing them in a sustained and slow manner. Additionally, immune modulators such as interleukin-15, interferon-γ or interleukin-2 in therapeutic hydrogels have been shown to enhance anticancer treatment efficacy without host immune system interference [31,37]. Hence, performing in situ chemotherapy via IMHs that possess antitumor properties can lead to enhanced outcomes while decreasing side effects (cytotoxicity for healthy tissue) (Figure 1C) [38].

### 1.3. Diabetes Mellitus

Inability to either produce enough insulin or have proper insulin-based signaling causes diabetes mellitus, which is characterized by high blood glucose levels. As such, the patients need to be constantly monitored for their blood glucose levels. Currently, several implant devices are commercially available that can monitor blood glucose levels, using conventional approaches and materials. Nevertheless, the foreign body response and consequent fibrosis incidents hinder implantable technologies’ full potential for in vivo applications [39,40]. IMHs with their potential to control foreign body responses and fibrosis can avoid such immune responses and offer a better performance of monitoring implantable devices [41,42]. One such example is that of supramolecular peptide-based hydrogels such as D-Gly-Phe-Phe-Tyr tetrapeptide which have been used both as insulin depots and immune adjuvants [43]. Additionally, hydrogels with antifouling properties can accelerate the healing of chronic wounds caused by advanced diabetes. Furthermore, by regulating the composition of cationic chitosan and anionic dextran, IMHs that can stimulate fibroblast migration, granulation tissue development, and angiogenesis can be created to further exploit these hydrogels for additional biomedical applications [44]. Additionally, one can take advantage of the drug loading abilities of IMHS by encapsulating them with anti-inflammatory drugs such as paeoniflorin [27] or growth factors such as fibroblast growth factor (bFGF) (Figure 1D) [45], whereby they can ameliorate inflammation through modulation of macrophages and epithelialization of wounds.

### 1.4. Sustained Release Drug Delivery 

Biomaterial-based drug delivery has been widely explored for immunotherapy applications [16]. Sustained-release systems offer several chemical and physical advantages that have been utilized to enhance the therapeutic window in addition to immunomodulation in drug administration. Using biodegradable materials for localized sustained release of encapsulated drugs is a frequently employed technique to reduce high and frequent dose intakes [46,47]. Likewise, drug clearance and degradation, two critical parameters of soluble drug delivery, can be reduced by biomaterial encapsulation [36,48]. Different aspects of biomaterials play an important role in these phenomena. Drug release is regulated by drug diffusion, hydrogel degradation, swelling, and environmental or chemical stimuli [32,49]. For example, through the oxidation of biomaterials and by changing the hydrophilicity, one can stimulate a morphological transition in micelles resulting in desired drug release using these platforms (Figure 1E) [50]. Alternatively, encapsulated cells and micro-tissue transplants can be engineered to mitigate immune response to graft rejection and augment treatment efficacy [51,52].

## 2. Types of Hydrogels Used for Immunomodulation

**Figure 2:**
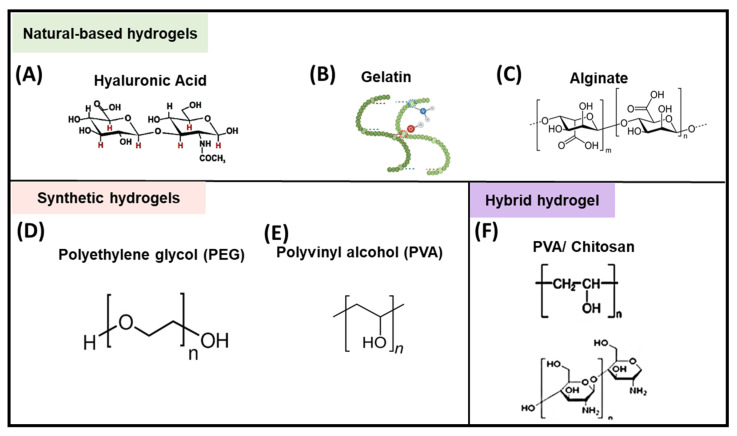
Different types of immunomodulating hydrogels. (**A**) Structural formula of hyaluronan disaccharide unit, (**B)** Molecular structure of gelatin (redrawn from the original article [57]), (**C**) Molecular structure of alginate, (**D**) Molecular structure of PEG, (**E**) Structural formula of PVA (**F**) Molecular structure of hybrid hydrogel composed of PVA/Chitosan.

Ranging from natural to synthetic polymers, there is a broad choice of molecules for hydrogel synthesis [8,9,11]. Synthetic polymers such as polyethylene glycol (PEG), provide more stability regarding structural and mechanical properties of the hydrogels [10,11], whereas natural polymers such as gelatin, collagen, cellulose, xanthan, alginate, hyaluronic acid (HA) and dextran, tend to confer better biocompatibility and reduced immunogenicity, improved cell adhesion, and proliferation [8,10,11,53,54,55]. The selection of the polymers depends on the application, the biomolecules or drugs to be encapsulated, and the target tissue. Additionally, hybrid hydrogels have been proposed that combine natural and synthetic polymers and bring out the respective material properties that make them very unique in their applications [11,56]. In this section, we will discuss some examples of natural, synthetic, and hybrid polymeric hydrogels that are used for immunomodulation purposes (Figure 2).

### 2.1. Natural Hydrogels

#### 2.1.1. Hyaluronate/Hyaluronic Acid (HA)

Hyaluronate is a glycosaminoglycan (GAG), a key component of extracellular matrix (ECM) which is present in the majority of tissues but mainly in connective, epithelial, and nerve tissues (Figure 2A) [54]. HA can modulate cell migration, inflammatory response, and angiogenesis [9,54].

#### 2.1.2. Gelatin

Gelatin can be obtained from numerous animal sources such as swine [58], squid [8], fish [57], beef [59], and camel [60]. Gelatin has been conjugated with a wide range of immunomodulating molecules (Figure 2B). One such example includes a gelatin-based hydrogel with IL-10 and PG-E2 that has been used as an immunomodulatory coating for silicone 3D tracheal implants to prevent granuloma formation. This formulation demonstrated an upregulation of M2 macrophages and low levels of proinflammatory cytokines, thereby controlling the inflammatory response [55]. Furthermore, gelatin methacryloyl (GelMA) when combined with IL-6 resulted in double survival of skin allografts compared with IL-6 administered alone [61].

#### 2.1.3. Alginate

Alginate is an alga-derived anionic natural polymer. In the presence of Ca^2+^ ions and mature dendritic cells, alginate hydrogels have been demonstrated to attract immune cells to the site of injection, leading to a greater immune response against infection [10]. Moreover, alginate gel formulations with calcium carbonate (CaCO_3_) have displayed faster-wound healing by macrophage polarization to the M2 phenotype when applied to skin flap ischemic wounds (Figure 2C) [56]. 

### 2.2. Synthetic Hydrogels

PEG is a synthetic polymer that is used as a constituent in healthcare-related products. It has also been used for hydrogel synthesis and applications. Several formulations with PEG have demonstrated biocompatibility and effectiveness for cancer immunotherapy when combined with growth factors, cancer antigens, and antigen-presenting cells [10,62]. Additionally, PEG with TGB-ß1 and IL-10 has also been effective for immunosuppression by decreasing dendritic cell maturation [10]. Furthermore, PEG-based hydrogels loaded with human mesenchymal stem cells (hMSC) and IFN-γ have also shown accelerated colonic mucosal wound healing in immunocompetent and immunocompromised mice [63]. PEG and other types of synthetic hydrogels such as polyvinyl alcohol (PVA) and polylactic co-glycolic acid (PLGA) have been useful for cartilage tissue engineering as well (Figure 2D) [24].

### 2.3. Hybrid Hydrogels

To improve mechanical properties as well as biocompatibility, natural and synthetic hydrogels can be conjugated together. For example, it has been demonstrated that gelatin derived from squid cartilage when combined with methacrylated HA (HAMA) and crosslinked with PEG has improved mechanical properties. Furthermore, this hybrid hydrogel is capable of tissue restoration as well as inducing an immunomodulatory response to neutrophils and anti-inflammatory macrophages, resulting in cartilage formation [8]. Also, an injectable hydrogel composed of PEG and vascular endothelial growth factor (VEGF) showed cardioprotective properties and improved vascularization in a rat model [9]. Additionally, hydroxyapatite combined with an hMSC hydrogel and a 3D-printed model for bone regeneration has demonstrated enhanced osteogenesis, macrophage regeneration, and angiogenic properties [64]. These are only a few examples of the diversity that can be achieved by combining natural and synthetic polymers in order to obtain better hydrogel properties (Figure 2F).

In the next sections, we discuss immunomodulating strategies and different molecules that might be potential new candidates to explore when designing IMHs. We will briefly discuss strategies that can be used on a systemic level or localized together with candidates for immunomodulation to be used for specific diseases.

## 3. Immunomodulation Strategies

### 3.1. Systematic Strategies

**Figure 3:**
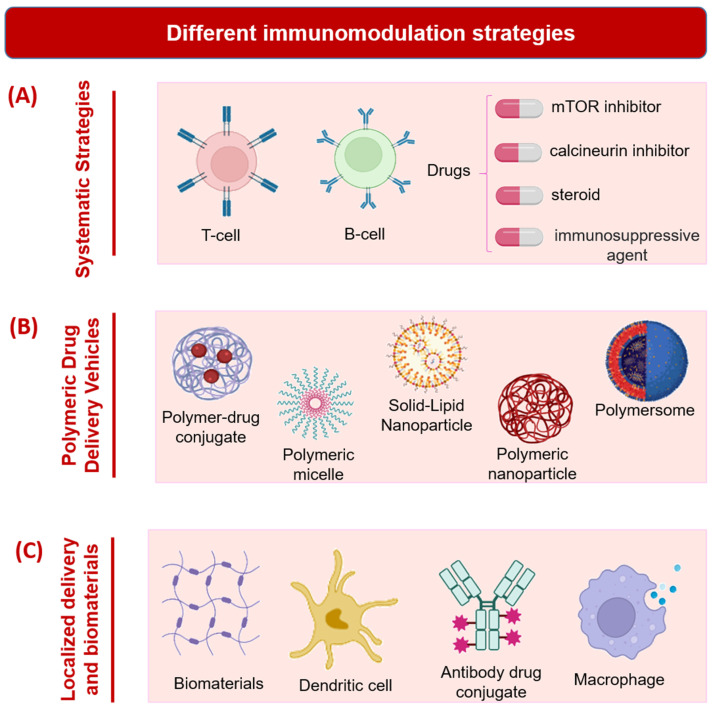
Different immunomodulation strategies. (**A**) Systematic strategies. (**B**) Polymeric drug delivery vehicles. (**C**) Localized delivery and biomaterials. Schematics prepared using Biorender.

Tolerogenic therapy and systematic immune suppression following allogeneic tissue or cell transplantation are often needed to avert pernicious autoimmune events [65]. These same issues also need to be considered when using IMHs for biomedical applications. T cell activation, effector cell differentiation, immunological priming, and effector T cell trafficking have all been documented to occur with the clinical introduction of scaffolds, devices, implants, and any materials which are recognized as foreign by the immune system [66]. Defective T cells also produce rigorous, unwanted autoimmune cycles leading to antigen-specific inflammation, which resemble phenomena occurring in the spinal cord and other traumatic injuries. Indeed, T cells are often the key players or targets in adaptive immune system remodeling and their modulation can be achieved by alterations that are made to the innate immune system [66]. Currently, blocking antibodies or immunosuppressants of different drug classes including, but not limited to, sirolimus (mammalian target of rapamycin, or mTOR inhibitor), tacrolimus (calcineurin inhibitor), and prednisone (steroid) are used to dampen auto- or alloimmune responses (Figure 3A) [67]. However, these often produce a significant decline in anti-infectivity and leukocyte-mediated tissue regeneration at the site of an injury. In one study, administrating targeting molecules or substances systemically has been shown to aid in immunomodulation and antigen tolerance [66]. For instance, by the intravenous route, Tregs have been utilized to reduce critical immunity caused by isoantigens arising from islet graft transplantations [68]. This occurs due to their counteractivity to effector T cells that are antigen-specific. Mesenchymal stem cells, combined with mycophenolate mofetil (MMF), have also been delivered into the veins of mice after transplantation surgeries, and their function is to increase the number of T helper 17 cells, which MMF interacts with to produce effector T cells [69]. In fact, systematically rather than locally administered MSCs lead to the higher presentation of Tregs throughout the body and reduce the potential for inflammation following allograft implantation [66].

### 3.2. Polymeric Drug Delivery Vehicles

In another strategy for the modulation of the immune response, polymeric nanoparticles have been systemically introduced in many instances to emulate apoptotic cell fragments (Figure 3B). In one study, non-biodegradable, carboxylated and myelin integral membrane protein-conjugated polystyrene (PS) particles (500 nm) were fabricated and intravenously administered into mice with autoimmune encephalomyelitis [70]. With the (139–151) protein epitope, the polymer nanoparticles prevented or slowed progression of the brain and spinal cord inflammation associated with this condition. Moreover, studies investigating PS nanospheres, showed that those with a larger size (500 nm^−1^ µm) and with an anionic charge on their membranes were more likely to interact with macrophage receptors with collagenase structure (MARCO), which determines the degree of diminished immunogenicity [70]. Other strategies attempted for treating autoimmune encephalomyelitis have used poly (lactide coglycolide) (PLG) nanoparticles as carriers of different substances, such as antigen-specific peptides, and immunosuppressant drugs, which can transiently inhibit the activation of T helper 1 and 17 cells, monocytes, and CD4+ and CD8+ T cells, among others [70].

### 3.3. Localized Delivery and Biomaterials

Systemic strategies have been shown to be effective, although potential antagonistic responses, including intolerances in the gastrointestinal tract, secondary immune side effects, or the evolution of antibiotic-resistant microbes could occur [66]. The local delivery of proteins, genes, cells, or biomaterials could help to avoid these potential side effects and still enable immunomodulation in the absence of circulating factors. Moreover, there are advantages of using local over systemic approaches, especially when targeting molecules or antibodies that are not incorporated into the formulation being administered, of improved tissue specificity and functional modulation [71]. In one well-characterized phenomenon that occurs in the central nervous system, microglia and astrocytes co-operate to reduce inflammation caused by injury and eliminate excitotoxicity [72]. The local delivery of immunomodulators could promote direct interaction with these cells and any associated inflammatory cells or molecules. Indeed, design considerations often implicate the interacting immune cell populations that will become affected by a locally administered immunomodulating agent. Often, these populations are somewhat distinct in their functions or responses across tissue types, despite falling into the same categories that encompass the traditional immune cells, such as the macrophages, lymphocytes, and neutrophils (Figure 3C). For example, the liver and epidermis, respectively, contain specialized Kupffer cells or hepatic macrophages and Langerhans cells, which have a macrophagic cell lineage as well as dendritic cell properties, that are situated in the outer skin layers [73,74]. As elaborated above in the discussion on systemic cell delivery, stem cells, especially MSCs, are excellent for re-configuring immune system functions (e.g., by altering macrophage polarization states, changing immune cell phenotypes, or reducing inflammation). Their localized transplantation can be utilized to achieve good clinical outcomes in terms of tissue regeneration and immunomodulation. This is accomplished by direct interactions between native and transplanted cells or by the transmission of cell signaling proteins or molecules, such as cytokines and hormones, that broadcast messages between interacting cell types. Tregs have also been transplanted singularly or together with PLG biomaterials in order to inhibit effector T cell and dendritic cell responses and to prevent implant rejection by the immune system [75]. They function by secreting proteins including the cytokines IL-10, galectin-1, and TGF-β, or by interacting with the host cells through several immune-regulating protein-mediated pathways [76]. An interesting study investigating locally transplanted Tregs demonstrated that systemic protection (in distal areas) could only be achieved via regional administration of these cells [75]. Moreover, when immune aversion, rather than modulation, is desired in tissue engineering applications, embryonic stem or other fetal cells which are deficient in major histocompatibility complexes I and II can be transplanted [77,78].

This leads to a discussion on biomaterials and tissue growth scaffolds such as IMHs as modifiable substrates that can be used locally to prevent autoimmunity from occurring or lessen the extent or severity of an autoimmune response. Regenerative medicine utilizes biomaterials and hydrogels in order to internally support the growth, proliferation, and differentiation of autogenous or allogeneic cells, including stem cells; to provide material or a base for host tissue complexation and integration, and to restore functional performance in damaged organs or tissues [79]. Properties of biomaterials, including hydrogels, therefore must be evaluated in order to determine the optimal structure, porosity, surface features, chemical composition, and potential for functionalization with cells, nanoparticles, proteins, genes, or other molecules [80,81]. These properties influence the eventual performance, including the immunomodulation, of hydrogels and other biomaterials in vivo. An example of this would be the prevention of lymphocyte adhesion onto biomaterials by attaching the Fas ligand to them, which can bind the Fas receptors on lymphocytes and deactivate them [82,83]. In alternate approaches, structural porosity, as well as fiber alignments and diameters in electrospun scaffolds, have been optimized to minimize fibrous capsule formation and improve host/implant assimilation [66]. These properties have been determined to influence the release of cytokines and other biomolecules by host immune cells. 

The hydrophobicity of a material can also modulate the behavior of immune cells, as more hydrophilic surfaces tend to recruit few monocytes and macrophages overall [84]. Foreign-body giant cells, which are conglomerates of macrophages, are also less likely to become activated when hydrophilic materials are implanted [85,86].

## 4. Immunomodulating Molecules

**Figure 4:**
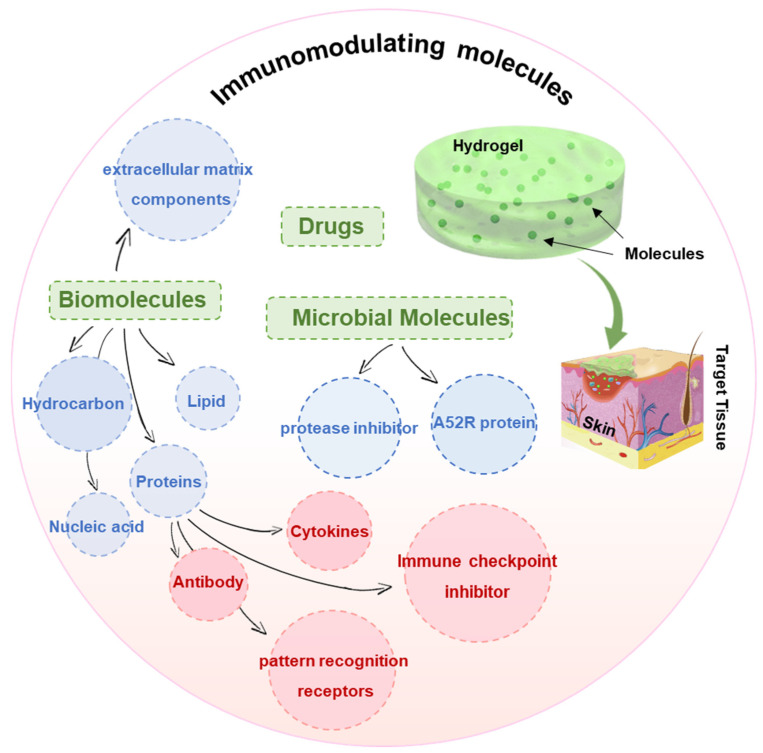
Scheme of different types of immunomodulating molecules.

The molecular mechanisms that elicit an immune system response are complex and multi-faceted as they involve the biological presentation of various substances including biomolecules, drugs, proteins (such as cytokines, antibodies, immune checkpoint inhibitors, and pattern recognition receptors) and peptides, nucleic acids, and extracellular matrix components. These substances can be naturally derived from organisms, even from bacteria, fungi, and parasites, or synthetically fabricated. Other constructs that are made up of molecules, including genes and cells, are also applicable to immunomodulatory delivery, although this section will specifically be aimed at describing the molecules that activate an immune response (Figure 4). Many of these can be incorporated into hydrogels using traditional physical and/or chemical methods. These molecules may be comprehensive or specific in their ability to alter immune functions depending on their composition, structure, and biological activity. Moreover, molecular targeting to treat certain cancers, inflammatory conditions, and autoimmune disorders, including diabetes, is also possible by the regulation of immune system components.

### 4.1. Antigen Models

The properties of the extracellular matrix (ECM) constitute one of the most important parameters in the regulation of an immune response. ECM constitutes different factors such as laminin, collagen, fibronectin, and proteoglycan, which affect cell function and immunogenicity. Furthermore, ECM contains bioactive agents such as antigens, cytokines, and other immunomodulatory molecules. The interaction between ECM components and antigen molecules has been shown to positively affect tissue regeneration, healing, and therapeutic applications [87,88,89]. Proof of concept studies in immunomodulation have utilized antigen molecules in order to assess strategies in vaccination efficacy, though many of their effects are only partially translated to human studies [90]. These model antigens include ovalbumin from chicken eggs, bovine serum albumin, and dinitrophenol, which are classically recognized as allergenic proteins or small molecule antigens, respectively. In turn, experimental molecules such as α-galactosylceramide, which is a natural killer T cell ligand that functions as an adjuvant, can also be co-administered with the model antigens to increase the potency of the complete formulation. Besides producing a potent immune response by nasal spray application in BALB/c and C57BL/6 mice, this adjuvant and antigen complex has been proven effective in combatting EG7 lymphoblasts cells [91].

### 4.2. Immunomodulating Molecules in Cancer

Immunotherapy has become a promising technique to treat cancer by using localized immune niches such as hydrogels, which can act as three-dimensional biomedical cargo carriers and can regulate the tumor microenvironment for better therapeutic output [38]. In immunomodulation, proteins, and small molecules such as antibodies and immune checkpoint inhibitor drugs, which are often composed of antibodies as their active ingredients, are used. For example, in 2011, the immunotherapeutic ipilimumab, a monoclonal antibody and checkpoint inhibitor, was approved by the FDA for use in melanoma and other cancer patients [92]. These checkpoint inhibitors primarily govern the native T cell processes after their administration [93]. For instance, the co-inhibitory molecule cytotoxic T lymphocyte antigen 4, or CTLA-4, which is found in high amounts in regulatory T cells, is blocked by ipilimumab or tremelimumab from binding B7 protein, thus allowing the elimination of tumor cells by T cells [92,94].

Advanced checkpoint inhibitors have also been produced to target apoptotic cell death proteins and ligands, including PD-1 (programmed cell death protein 1), a receptor of the B7 family, and the associated ligands PD-L1 and PD-L2 (programmed death ligands 1 and 2), especially in cancer immunotherapy [92]. Nivolumab, a G4 immunoglobulin, has been shown to be safe and effective as a PD-1 inhibitor, and other molecules that are undergoing evaluation for their anti-PD1 activity include pembrolizumab and pidilizumab humanized monoclonal antibodies, as well as AMP-224 protein [95,96,97]. Unlike ipilimumab, nivolumab was not dose-dependent in terms of the efficacy return following administration, although both drugs demonstrated anti-cancer responses only after prolonged treatment, during which time there was a stable progression of the disease state before amelioration occurred [92]. Atezolizumab and BMS-936559 are, similarly, antibody treatments that target the PD-L1 ligand in cancer cells, and in other instances, checkpoint inhibitors such as lirilumab are being investigated for their ability to bind alternate T and natural killer cell receptors, other than CTLA-4 or PD-1 [98,99]. Many of these molecule drugs can be used in consortium with chemotherapeutics in designed IMHs to achieve a potent anti-cancerous effect.

### 4.3. Immunomodulating Molecules in Autoimmune Diseases

There are more than 80 different autoimmune diseases, with the most common ones being type-1 diabetes, rheumatoid arthritis, multiple sclerosis, and systemic lupus erythematosus [100]. Complementary strategies have been applied in the immunomodulation of these and other autoimmune diseases. In the case of implantable biomaterials, a common problem with implications for their effectiveness is an immune response from the host [55]. Many factors such as physicochemical and biocompatible properties, and the location of an implanted material can determine the severity of the immune response [101]. To boost diabetic-wound healing, a photocurable methacryloxylated silk fibroin hydrogel (Sil-MA) system co-encapsulated with metformin-loaded mesoporous silica microspheres (MET@ MSNs) and silver nanoparticles (Ag NPs) was developed. Sil-MA-based hydrogel showed a sustained and controlled release of the Ag nanoparticles. Moreover, this hydrogel platform inhibited the formation of neutrophil extracellular traps, which induces pro-inflammatory factors. Due to its ability to modify the immune microenvironment, this IMH system promoted fibroblast migration and endothelial cell angiogenesis [102].

Other strategies and approaches have also been developed and applied in the immunomodulation of autoimmune diseases. In type I diabetes, the preservation of beta cells is a primary objective for which drugs and therapies have been developed [103]. One approach utilizes anti-CD3 antibodies in order to slow beta cell deterioration since the CD3 receptor is involved in a rigorous autoimmune response in diabetic patients [104]. This tactic delayed the progression of type I diabetes by just one year, perhaps, alternate strategies including an anti-CD3 booster are needed for the long-term. Moreover, cytokine release syndrome, low blood platelet counts, and Epstein-Barr viral reactivation were serious side effects associated with the CD3 antibody treatment approach [103].

To overcome these challenges, other approaches undergoing empirical evaluation include the nonmyeloablative transplantation of autologous stem cells, autoantigen treatment, and vaccination with glutamic acid decarboxylase [103]. Autologous stem cell transplantation is risky and can lead to high rates of treatment-related mortality, as discerned from previous applications to other autoimmune conditions, hence other methods may be preferred [103,104,105]. For instance, in the autoantigen approach, one direct influencer of beta cell functions is insulin, which has been subcutaneously administered to pre-diabetic patients as a preventative measure [106]. However, the preventative success of insulin injections was not substantiated by clinical trials, despite the efficacious use of insulin in treating type I diabetes. Other indirect autoantigens are heat shock proteins, or their synthetic peptide counterparts such as DiaPep277, which has been used to preserve insulin control and secretion by the beta cells without producing adverse side effects in adult patients with type I diabetes [107]. Glutamic acid decarboxylase, a metabolic rate-determining enzyme for the process of glutamate to gamma-aminobutyric acid conversion, has also been shown, like autoantigens, to cause a Th1 to Th2 transition of T cells [108]. A vaccine, Diamyd, containing this enzyme and the aluminum hydroxide adjuvant has demonstrated the potential to prevent diabetes in numerous clinical trials in the United States and Europe [109].

### 4.4. Microbial Molecules as Immunomodulating Agents

Microbiota-derived molecules have also been used in immunomodulation, and they will be briefly described here [110]. Increasingly, type I and type II diabetes are linked to intrinsic changes in gut microbiota, which may release smaller quantities of short-chain fatty acids, such as butyrate [110,111]. Diminished butyrate conversion from lactate has been correlated with reduced regulatory T cell generation and a weakened suppression of the autoimmune system [112].

One recent study has described hyaluronic acid (HA) hydrogels as having triple biological activities of being antimicrobial, immunomodulatory, and capable of acting as a miRNA delivery agent. MiRNA with antibacterial and anti-inflammatory properties was loaded into these hydrogels. Furthermore, the HA hydrogel was fabricated with polyarginine, which reduced the inflammatory response of lipopolysaccharide-stimulated macrophages [113].

In other instances, immunomodulatory molecules derived from pathogenic or non-pathogenic microbes have been applied in acquiescing inflammatory conditions [114,115]. For instance, a protein of the vaccinia virus has been demonstrated to interrupt the complement system cascade that occurs following the implantation of a foreign body, and also to avert immune system-induced damage to the central nervous system [114,116]. Another example is of a protease inhibitor released by the myxoma virus, stress-associated endoplasmic reticulum protein 1, which is anti-inflammatory in patients with acute coronary syndrome [114,117]. Numerous cytokines, chemokine homologs, chemokine binding proteins, and cell signaling molecules released by viruses, bacteria, and fungal species have also shown efficacy in blocking infections, suppressing inflammation, and inhibiting chemokines, for example, the A52R protein, which is also a product of the vaccinia virus, reduces inflammation by inhibiting the generation of NF-kappaB proteins through toll-like receptor mechanisms [118].

## 5. Future of Immunomodulating Hydrogels

The future of IMHs lies in healing skin infections and wounds, treating various forms of cancerous tumors, and more through regulating the various immune effects of different regenerative and implant platforms. While promising hydrogel properties and results are being found regarding each of these goals, many challenges still lie ahead including bringing these hydrogels to clinical trials and developing improved biomaterials for even better immune regulation.

### 5.1. Skin Infections

Disruption of the tissue microenvironment due to various reasons such as loss of extracellular matrix, inflammation, and impaired angiogenesis is one of the most important therapeutic targets in wound healing. IMHs show incredible promise in healing skin infections. In a study on dendritic hydrogels, the hydrogels were able to stop bacterial infections induced by drug-resistant bacteria by enhancing the expression of antimicrobial peptides in skin cells [64]. Hydrogels’ ability to stimulate macrophages has also been used to control the inflammatory response over time, periodically activating a certain integrin for 72 h in order to sustain an anti-inflammatory M2-macrophage response [21]. These anti-inflammatory and anti-bacterial properties along with biocompatibility and fast degradation signal a future for immunomodulating hydrogels in treating skin infections. Moreover, IMHs are a promising platform for overcoming many limitations related to wound dressing treatments for skin infection that can enhance the remodeling of existing tissues, which can be widely used in healing wounds caused by conditions such as burns, cuts, and diabetes. Here, we briefly discuss specific medical conditions where IMHs were used in pre-clinical studies and outline the future opportunities and challenges IMHs face in immunomodulation strategies for grafts and regenerative medicine.

### 5.2. Tissue Regeneration and Wound Repair

In the field of tissue regeneration, IMHs have been used in 3D printed hybrid scaffolds to stimulate macrophages to release growth factors and promote bone and vessel formation [119]. The squid cartilage type II gelatin (SGII) and HA (SGII/HA-DN) hydrogel have also been used to regulate the local microenvironment and induce cartilage regeneration in vivo [8]. The immunoregulatory properties of hydrogels being used as tissue scaffolds and to induce cartilage regeneration demonstrate the potential of IMH use in tissue regeneration applications. An area for future development could be controlling macrophage polarization, which will allow further advancement in the management of regenerative medicine by using hydrogel-based biomaterials.

Hydrogels with encapsulated hMSCs and tethered immune proteins (IFNγ) were used to accelerate the healing of colon wounds in mice with normally functioning and compromised immune systems [63]. Another study using hydrogels to deliver these IFNγ proteins showed that immunomodulatory bone marrow genes were expressed for longer periods compared to 2D tissue culture [120]. Nanoparticle-laden hydrogels were also used to accelerate wound healing through their adhesive ability to cover an entire wound and promote skin vascularization [121]. Iwhile Biomaterials containing cryogel/hydrogel have shown antimicrobial properties, exudate sorption, and immunomodulation activity in diabetic skin wounds by increasing M2-macrophage population level [122]. Another method of increasing and extending the therapeutic effects of using hydrogels is by recruiting endogenous immune cells, which may produce fewer side effects than directly delivering growth factors [21]. Other methods of modulating immune responses for wound healing include differentiating T cells, expressing inflammatory activation markers, and delivering bioactive molecules via different routes [123,124]. This multitude of avenues to enhance and accelerate wound healing show that IMHs have strong potential in wound-healing applications.

### 5.3. Prevention of Tumor Metastasis

In addition to skin and wound repair, IMHs have been used to prevent tumor metastasis and suppress/eliminate tumors. The approaches of hydrogel-mediated immunomodulatory agents to the management of antitumor immune responses and local tumor inhibition have recently attracted considerable attention in the use of treatment types such as immunotherapy, chemotherapy, radiotherapy, and phototherapy. An injectable hydrogel was used to block the ARG1 pathway and reverse the ARG1 immunosuppressive environment, allowing for enhanced immunotherapy. This suppressed tumor growth and prevented pulmonary metastasis [39,125]. A bioadhesive hydrogel was also demonstrated to prevent tumor metastasis by immobilizing the solid tumor. This provided an immune response site after injection and created immunomodulation in a localized area [38]. This was also demonstrated in a melanoma mouse model. Hydrogels have also been used to deliver dendritic cell vaccines and inhibitor medication for cancer [126]. These immunoenhancing effects, immune response site creation, and use of hydrogels as a mechanism to deliver other healing materials all point to the future potential of IMHs in cancer treatment and in the prevention of tumor metastasis.

### 5.4. Future Steps

The future of IMHs entails continued experimentation to bring these promising properties to patients. This will involve creating an ideal biomaterial and overcoming the challenges that come with fabricating these hydrogels and making them ready for clinical trials. Part of the challenge is that immune cells are extremely sensitive to their microenvironment, and the immune system has an innate response to foreign material, no matter how biocompatible it is. In addition, immune cell behavior is dependent on individual patients’ medical history. These factors enhance the difficulty of preparing hydrogels for clinical trials [120,127]. Criteria for developing an ideal biomaterial for immunomodulation and successful clinical trials include having an optimal degradation time, avoiding triggering an immune response, and demonstrating efficacy of immunomodulation. The use of MSCs with immunomodulatory properties and their immunophenotypic structure along with hydrogels may have great advantages not to cause a significant immune response. Another challenge to overcome is control over the release of bioactive molecules. Releasing molecules too quickly could result in immune tolerance, and releasing too slowly could result in a lack of activation [127]. Some strategies proposed to deal with these challenges include the design of hydrogels that respond to controllable stimuli and creating small platforms for chronic wounds. Incorporating flexible electronics has also been proposed to monitor wounds and control bioactive molecule release [123]. Once the challenges of material development for clinical trials are addressed, IMHs will offer tremendous opportunities for various medical applications in healing skin infections, wounds, and tumors, as well as regenerating tissues.

## Data Availability

Not applicable.
